# Neuropathic Pain Comorbid Cognitive Impairment and Vitamin D: A Narrative Review From Mechanisms to Clinical Management

**DOI:** 10.1002/fsn3.72440

**Published:** 2026-10-21

**Authors:** Nan Zhao, Yang Yang, Wanqiu Yu, Yuhang Zhu, Zhaoqiong Zhu

**Affiliations:** ^1^ Department of Anesthesiology Affiliated Hospital of Zunyi Medical University Zunyi China; ^2^ Department of Anesthesiology Hospital of Stomatology, Zunyi Medical University Zunyi China; ^3^ Early Clinical Research Ward Affiliated Hospital of Zunyi Medical University Zunyi China; ^4^ Key Laboratory of Brain Function and Brain Disease Prevention and Treatment of Guizhou Province Affiliated Hospital of Zunyi Medical University Zunyi China

**Keywords:** cognitive impairment, dietary supplements, neuralgia, neuroprotection, vitamin D

## Abstract

Neuropathic pain (NP) is not merely simple persistent pain but a complex pathological condition mediated by dysregulation across multiple systems, including the neuro‐immune‐endocrine axis. As a high‐intensity endogenous stressor, persistent pain signals can deplete cognitive resources and trigger neuroinflammation and central sensitization, leading to cognitive impairment (CI) characterized by attention deficits and impaired working memory. Currently, mainstream pharmacological treatments for NP carry risks of addiction and may exacerbate CI. Therefore, there is an urgent need to develop synergistic intervention strategies that address both pain management and cognitive protection. Given that NP and CI share multiple pathological pathways, identifying interventions capable of simultaneously targeting multiple mechanisms has become a research hotspot. Vitamin D, a secosteroid hormone with both neuroprotective and immunoregulatory functions, is closely associated with an increased risk of NP and CI when deficient. Existing evidence suggests that Vitamin D may regulate the shared pathological mechanisms of these two diseases through multiple pathways, thus serving as a potential adjunctive strategy worthy of further investigation to alleviate the intertwined vicious cycle between NP and CI. This paper systematically reviews the shared pathological mechanisms between NP and CI, summarizes the latest research progress, and explores the multi‐target mechanisms of Vitamin D in intervening in NP‐CI comorbidity, aiming to promote the application of this safe and cost‐effective nutritional strategy in dietary supplementations for NP‐CI comorbidity.

## Graphical abstract

Vitamin D is an essential fat‐soluble nutrient primarily obtained through cutaneous synthesis triggered by ultraviolet exposure and dietary intake. After systemic metabolism and activation, it binds to vitamin D receptors widely distributed in the central and peripheral nervous systems, exerting pleiotropic neuroprotective and regulatory biological functions. Preclinical and clinical evidence indicates that vitamin D and its receptor play roles in the onset and progression of neuropathic pain and cognitive impairment by modulating neuroinflammation, maintaining endocrine homeostasis, balancing brain networks, mediating epigenetic regulation, and targeting immune‐metabolic pathways of the gut‐brain axis. This review constructs a conceptual framework bridging basic research and clinical practice, aiming to provide a nutritional strategy perspective for clinical intervention of such neurological comorbidities.

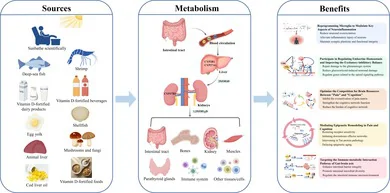

**Abbreviations**
1,25(OH)_2_D: 1,25‐dihydroxyvitamin D
1,25‐(OH)_2_D_3_
: 1,25‐dihydroxyvitamin D_3_
25(OH)D: 25‐hydroxyvitamin D
25(OH)D_3_
: 25‐hydroxyvitamin D_3_
5‐HT: 5‐hydroxytryptamine
7‐DHC: 7‐dehydrocholesterol
AD: Alzheimer's disease
AVP: arginine vasopressin
BBB: blood–brain barrier
BDNF: brain‐derived neurotrophic factor
CEN: central executive network
CI: cognitive impairment
CNS: central nervous system
DMN: default mode network
DPN: diabetic peripheral neuropathy
GABA: gama‐aminobutyric acid
GBA: gut‐brain axis
GLT‐1: glutamate transporter‐1
HDAC3: histone deacetylase 3
HPA: hypothalamic–pituitary–adrenal
LPS: lipopolysaccharides
NE: norepinephrine
NMDA: N‐methyl‐D‐aspartate
NP: neuropathic pain
PARP1: poly (ADP‐ribose) polymerase‐1
PHN: postherpetic neuralgia
PNS: peripheral nervous system
PVN: paraventricular nucleus
RCTs: randomized controlled trials
ROS: reactive oxygen species
SCFAs: short‐chain fatty acids
SCN: suprachiasmatic nucleus
SN: salience network
TPH: tryptophan hydroxylase
UVB: ultraviolet B
VDBP: vitamin D binding protein
VDD: vitamin D deficiency
VDR: vitamin D receptor
VDS: vitamin D supplementation

## 1 Introduction

Neuropathic pain (NP) is a pain syndrome directly caused by damage or dysfunction of the somatosensory nervous system and is also a common form of chronic pain. Its pathophysiological mechanisms are complex, involving multiple aspects such as peripheral and central sensitization, abnormal neural plasticity, and dysfunction of descending inhibitory systems (Ghazisaeidi et al. 2023). As a global public health issue, NP affects approximately 7% to 10% of the general population, often resulting in functional impairments and reduced quality of life, thereby imposing a significant socioeconomic burden (Van Hecke et al. 2014; Baskozos et al. 2023). The clinical manifestations of NP are highly heterogeneous, primarily characterized by spontaneous burning pain, electric shock‐like pain, or hyperalgesia, which significantly increases the difficulty of individualized treatment. During disease progression, multiple comorbidities often occur, among which cognitive impairment (CI) is particularly concerning (Gong et al. 2025). Epidemiological data indicate that the incidence of CI in patients with NP ranges from 11.4% to 31.7%, which is significantly higher than that in the general population. CI affects multiple cognitive domains, including information processing speed, executive function, and overall cognition (Povedano et al. 2007; Nahin and DeKosky 2020; Ni et al. 2026). The impact of CI on NP patients is multidimensional: it creates a bidirectional vicious cycle with pain, hindering treatment and rehabilitation, and increases diagnostic and therapeutic challenges due to symptom overlap and shared neural pathways (Moriarty et al. 2017; Koyama et al. 2021). CI is a multifactorial neurodegenerative spectrum disorder that affects multiple brain regions and neural cells, encompassing phenotypes ranging from delirium and mild CI to dementia. Clinically, NP patients often report cognitive symptoms such as “brain fog” and difficulty concentrating, with imaging confirming the presence of hippocampal atrophy; in some cases, the volume of the hippocampus can recover after postoperative pain relief (Noorani et al. 2022). Conversely, patients with CI‐related diseases such as Alzheimer's disease (AD) may experience abnormal pain perception and response due to widespread neuronal and synaptic damage affecting brain regions involved in pain processing (Yao et al. 2025). Research has confirmed that NP and CI do not exist in isolation; they share multiple neuropathological mechanisms and exhibit complex bidirectional interactions, posing significant challenges for clinical management (Moriarty et al. 2017; Tyrtyshnaia et al. 2025).

Despite the increasing diversity of clinical interventions, current treatments still face numerous challenges: traditional therapies for NP, such as opioids and gabapentin, may exacerbate drowsiness and cognitive decline (Li et al. 2022), while cognitive‐enhancing drugs, such as cholinesterase inhibitors, have no direct effect on pain (Eldufani and Blaise 2019). Given that NP and CI share key pathological pathways—including neuroinflammation, neuroendocrine dysregulation, and brain network imbalance—finding interventions that can simultaneously target multiple pathways has become a research hotspot. Nutritional intervention strategies are increasingly valued in prevention and treatment due to their high safety, good compliance, and ability to maintain homeostasis over the long term (Cheng, Huang, et al. 2024). Vitamins, as essential micronutrients for human metabolism, play a crucial role in maintaining brain health (Cortés‐Albornoz et al. 2021; Freedman et al. 2022; Li, Chen, et al. 2023). Among them, Vitamin D has gradually become a research focus due to its key neurobiological effects related to neurodevelopment, protection, and immune regulation, as well as its widespread distribution in the central and peripheral nervous system (PNS) (Savran et al. 2025; Pietruszkiewicz et al. 2025). Studies have shown that Vitamin D deficiency (VDD) is closely associated with an increased risk of NP, exacerbation of pain, and accelerated progression of CI (Seetan et al. 2021). During the pathological progression of NP, VDD or abnormal signaling pathways can promote disease progression by exacerbating neuroinflammation, weakening neurotrophic support, and disrupting immune homeostasis (Zhang et al. 2024; Shi, Yang, et al. 2025). Vitamin D can improve learning and memory functions by reducing oxidative stress and inflammatory marker levels in hippocampal tissue, inhibiting the expression of inflammation‐ and apoptosis‐related genes, activating the Nrf2/HO‐1 signaling pathway, and blocking ferroptosis induced by hippocampal neuronal aging (Li, Cao, et al. 2023; Shah et al. 2026). In the context of comorbidity between NP and CI, VDD can synergistically exacerbate neuroinflammation, enhance oxidative stress, and weaken neurotrophic support, thereby serving as a potential risk factor driving disease progression (Alessio et al. 2021; Cui and Eyles 2022). Existing clinical evidence indicates that regular supplementation of vitamin D can effectively reduce NP severity in specific populations, improve mood disorders, and exert positive effects on executive function and memory (Chen, Cheng, et al. 2024; Pinzon and Angela 2025; Gilbody and Gilbody 2025). However, evidence in this field remains inconclusive: conclusions from observational studies and randomized controlled trials (RCTs) are not entirely consistent, and existing RCTs generally suffer from small sample sizes and considerable heterogeneity in intervention protocols. More importantly, there is still a lack of high‐quality RCTs specifically targeting populations with comorbid NP and CI, using both pain relief and cognitive improvement as dual primary endpoints. Although the current level of evidence is limited, considering the safety, cost‐effectiveness, and accessibility of vitamin D, its use as an adjunctive therapy for managing NP comorbid with CI still holds certain promise in clinical practice.

Taken together, accumulating evidence suggests a close association among vitamin D, NP, and CI; however, critical gaps remain in current research. On one hand, although CI as a common comorbidity of NP has garnered increasing attention, the potential role of vitamin D as a mechanistic interface between the two has yet to be systematically evaluated. On the other hand, current basic and clinical studies have predominantly investigated the independent regulatory effects of vitamin D on either NP or CI, with no studies yet adopting a comorbidity perspective to comprehensively examine vitamin D's modulation of shared pathological pathways between the two. Furthermore, the translational pathway linking molecular mechanisms to clinical management strategies remains unclear, and there is currently no academic consensus on vitamin D screening, supplementation protocols, or monitoring standards specifically for patients with NP comorbid with CI. Based on the aforementioned research background and existing gaps, this narrative review aims to provide an integrated perspective that systematically elucidates the potential role of vitamin D in NP‐associated CI. Specifically, we will first outline the shared pathophysiological mechanisms underlying NP‐related CI, as well as epidemiological and interventional clinical evidence linking vitamin D status to outcomes in NP and CI, thereby establishing an updated and comprehensive analytical framework. Finally, we will discuss the limitations of current studies and identify priority directions for future research, with the goal of offering theoretical and practical guidance for mechanistic understanding, biomarker discovery, and the development of precision intervention strategies for this comorbid condition.

## 2 Methodology

NP and CI with preclinical mechanistic studies and clinical evidence of Vitamin D nutritional intervention. Relevant literature was retrieved from major scientific databases such as PubMed and Web of Science to include studies published up to December 2025. The search strategy followed the PICO framework, employing a combination of Medical Subject Headings and free‐text keywords. The main search terms included “Vitamin D,” “Cholecalciferol,” “Cognition,” “Cognitive function,” “Cognitive impairment,” “Cognitive dysfunction”; as well as “Neuropathic pain,” “Chronic pain,” and “Neuralgia,” combined using Boolean operators (AND, OR). Regarding literature inclusion, the scope mainly covered original clinical studies (including RCTs, cohort studies, case–control studies, and cross‐sectional studies), preclinical research (including in vivo animal experiments and cellular/molecular mechanistic explorations), systematic reviews/meta‐analyzes, and high‐quality narrative reviews. Exclusion criteria comprised studies unrelated to the topic, duplicate publications, non‐peer‐reviewed conference abstracts or editorials, as well as studies with significant methodological flaws or insufficient sample sizes that undermine the reliability of conclusions. The literature screening process consisted of two stages: initially, titles and abstracts were reviewed for preliminary screening to exclude obviously irrelevant studies; subsequently, full texts of preliminarily included articles were thoroughly read to comprehensively assess methodological quality, data completeness, and mechanistic relevance. At the mechanistic exploration level, this review prioritizes studies focusing on inhibiting the cascade of neuroinflammation, maintaining neuroendocrine homeostasis, optimizing central metabolic resource competition, epigenetic remodeling, and the immune‐metabolic interactions of the gut‐brain axis (GBA). The aim is to deeply elucidate the potential molecular basis of the comorbidity between NP and CI, as well as the application potential of Vitamin D. It is important to note that, as a narrative review, this article does not employ a strictly systematic review or quantitative meta‐analytic approach. Instead, it qualitatively summarizes and thematically synthesizes the included literature, aiming to systematically explain the potential roles and mechanistic pathways of Vitamin D in alleviating pain and improving cognitive function from an integrative perspective of nutrition and pathophysiology. It should be noted that this article is a narrative review. The PICO framework, literature search and screening processes, as well as the inclusion/exclusion criteria employed herein, are solely used to construct the review framework, systematically organize relevant literature, and enhance the transparency and readability of the review. They are not intended to implement a full systematic review process, nor should they be interpreted as the methodology of a systematic review. The included studies vary in levels of evidence, and their findings should be interpreted cautiously in light of the respective study designs.

## 3 Pathological Mechanisms Shared by NP and CI

The high comorbidity rate of NP and CI is not coincidental but results from complex pathological interactions. Current research has shifted from single‐mechanism approaches to integrated hypotheses, constructing a multi‐level framework that spans from molecular to systemic levels and from peripheral to central systems. At the molecular level, chronic neuroinflammation and abnormal activation of glial cells release inflammatory mediators that sensitize pain pathways and impair synaptic plasticity, triggering imbalances in glutamate, gamma‐aminobutyric acid (GABA), monoamine neurotransmitters, and the neuroendocrine axis. This shapes a neurochemical microenvironment characterized by increased pain sensitivity alongside cognitive decline. At the macro level of brain networks, these changes lead to functional connectivity and dynamic reorganization of the default mode network (DMN), salience network (SN), and central executive network (CEN). Dysregulated interactions and failed compensations among these networks constitute the system‐level neurobiological basis of comorbidity. Upstream factors such as genetic susceptibility, chronic stress, metabolic disorders, and GBA communication disturbances provide foundational explanations for these multi‐level pathological changes from the perspective of homeostatic regulation. It is noteworthy that these mechanisms do not follow a simple linear progression; rather, they form a synergistic effect through positive feedback loops, collectively driving and maintaining a self‐reinforcing pathological cycle. Figure 1 presents a comprehensive overview of the five major pathological mechanisms underlying NP‐CI comorbidity, highlighting the shared biological targets between the two conditions. Its purpose is to provide theoretical insights for breaking the vicious cycle and identifying potential therapeutic intervention points.

**FIGURE 1:**
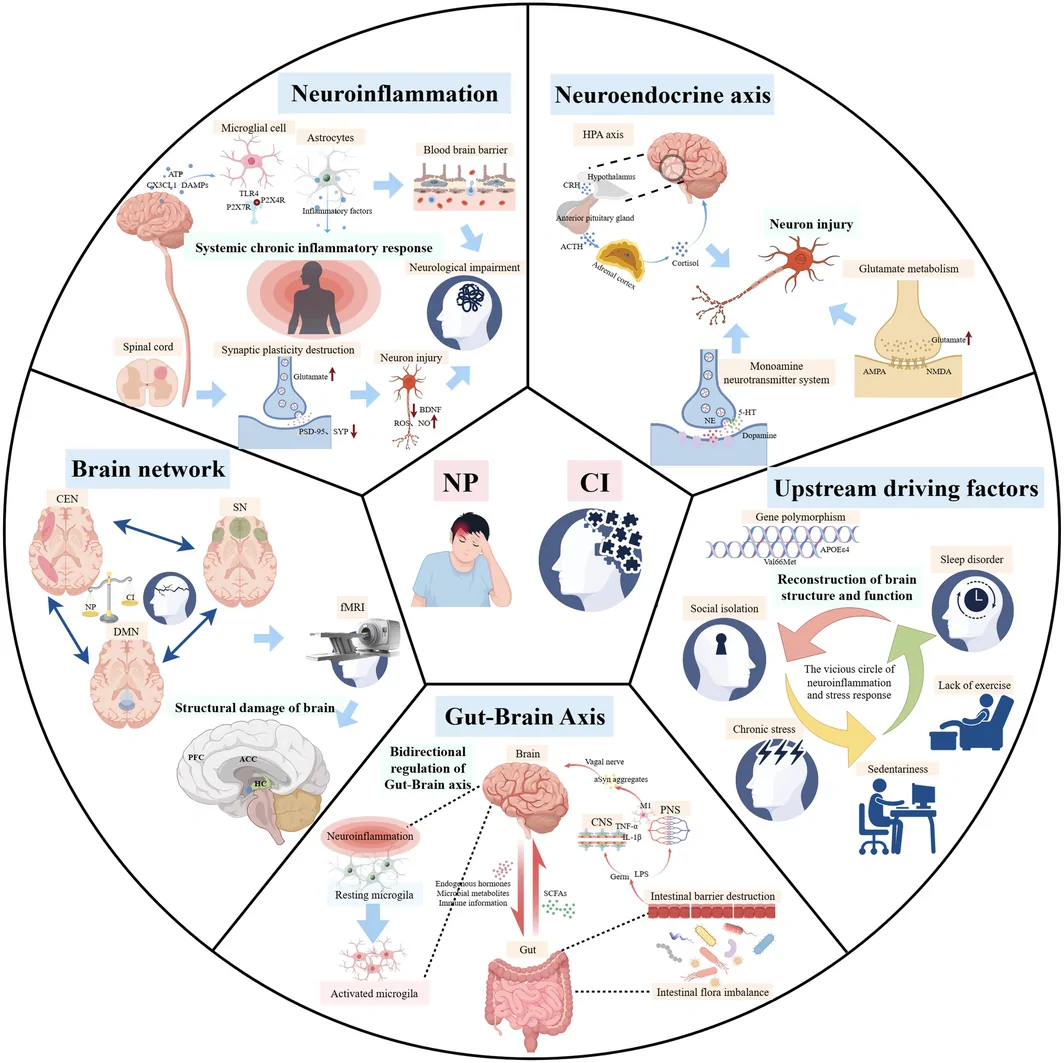
Panoramic pathological mechanisms of NP‐CI comorbidity. The high comorbidity rate of NP and CI is not coincidental but arises from a complex and intertwined shared pathological network. Neuroinflammation and abnormal activation of glial cells trigger and sustain inflammatory cascades, leading to excitotoxicity in neurons and impaired synaptic plasticity, while also collaboratively causing imbalances in neurotransmitter systems and endocrine axes. Molecular‐level disruptions and neuroendocrine dysregulation further disturb the dynamic balance among critical brain networks. Meanwhile, common upstream factors such as genetic susceptibility, chronic stress, metabolic abnormalities, and bidirectional communication disturbances of the gut‐brain axis continuously regulate and exacerbate neuroinflammation and brain network imbalances through epigenetic modulation, immune regulation, and metabolic pathways. The figure was created on https://www.Figdraw.com, has been authorized for use (copyright code: SOWRT8e33c). 5‐HT, 5‐hydroxytryptamine; ACC, Anterior Cingulate Cortex; ACTH, Adrenocorticotropic hormone; AMPA, Amino‐3‐hydroxy‐5‐methyl‐4‐isoxazolepropionic acid; ATP, Adenosine triphosphate; BDNF, Brain‐derived neurotrophic factor; CEN, Central executive network; CI, Cognitive impairment; CNS, Central nervous system; CRH, Corticotropin‐releasing hormone; DAMPs, Damage‐associated molecular patterns; DMN, Default mode network; fMRI, Functional magnetic resonance imaging; HC, Hippocampus; HPA, Hypothalamic–pituitary–adrenal; LPS, Lipopolysaccharides; NE, Norepinephrine; NMDA, N‐methyl‐D‐aspartate; NO, Nitric oxide; NP, Neuropathic pain; PFC, Prefrontal Cortex; PNS, Peripheral nervous system; ROS, Reactive oxygen species; SCFAs, Short‐chain fatty acids; SN, Salience network.

### 3.1 Inflammatory Cascade Mediated by Imbalanced Microglial Polarization

Peripheral nerve injury is a critical initiating event for central immune responses, with the imbalance in interactions between microglia and astrocytes constituting the core mechanism underlying the amplification of neuroinflammation and progression of neural damage. This process is regulated through specific molecular pathways, ultimately establishing the foundation for the mutual promotion of NP and CI comorbidity. After peripheral injury, the dorsal horn of the spinal cord releases adenosine triphosphate, chemokines, and damage‐associated molecular patterns (Pottorf et al. 2022). Microglia recognize the aforementioned signals via purinergic receptors (such as P2X4R and P2X7R) and Toll‐like receptor 4, rapidly polarizing from a resting state to a pro‐inflammatory phenotype and initiating inflammatory cascades (Wu et al. 2022). Activated microglia release pro‐inflammatory factors that recruit and activate astrocytes (Yao, Wang, et al. 2023; Müller and Di Benedetto 2025). The functional dysregulation of both cell types mediates pathological damage through multiple pathways: (1) Inflammatory amplification: Activated astrocytes produce large amounts of inflammatory mediators such as IL‐6, CCL2, and CXCL1, synergistically enhancing central inflammatory responses and disrupting the integrity of the blood–brain barrier (BBB), thereby promoting infiltration of peripheral immune cells (Zheng, Chen, et al. 2024); (2) Excitotoxicity: Astrocytes downregulate the expression of glutamate transporter‐1 (GLT‐1), leading to glutamate accumulation in the synaptic cleft, which directly damages neuronal structure and function (Dalkara et al. 2024; Gu et al. 2025); (3) Accumulation of inflammatory factors inhibits the synthesis and release of neurotrophic substances such as brain‐derived neurotrophic factor (BDNF), while simultaneously increasing the production of neurotoxic substances like reactive oxygen species (ROS) and NO, thereby accelerating neuronal apoptosis and aberrant circuit remodeling (Wang et al. 2025; Bo et al. 2025). The sustained interaction between glial cells leads to chronic local inflammation, driving the central nervous system (CNS) into a state of low‐grade chronic inflammation and establishing a microenvironment that underlies neuronal functional decline. The aforementioned molecular and cellular level changes ultimately integrate at the systemic level into a pathological chain reaction of “glial cell polarization imbalance‐neuroinflammation amplification‐ multiple neural injuries,” providing a key mechanistic support for the mutual promotion of NP and CI (Diotaiuti et al. 2024).

### 3.2 The Imbalance of Neurotransmitters and Endocrine Axes

The neuroendocrine axis circuit is a crucial pathway for maintaining homeostasis between the nervous and endocrine systems. This circuit begins with the regulation of hypothalamic neuroendocrine cells by CNS neurotransmitters, which then modulate the secretion of anterior pituitary hormones via the hypothalamic–pituitary portal system, driving hormone synthesis and release from downstream target glands. Besides directly acting on peripheral target organs, these hormones can also feedback to the CNS through the bloodstream or cross the BBB, directly influencing neurotransmitter synthesis and release. This forms a multilayered, bidirectional closed‐loop network involved in maintaining physiological homeostasis such as metabolism and stress response (Chang et al. 2025). In the progression of chronic pain, the monoaminergic neurotransmitter systems of norepinephrine (NE) and 5‐hydroxytryptamine (5‐HT) are primarily impaired due to depletion caused by sustained stress (Boccella et al. 2021; Mokhtar et al. 2025). Simultaneously, excessive glutamatergic excitation is manifested by enhanced phosphorylation of N‐methyl‐D‐aspartate (NMDA) receptors—particularly the Glutamate ionotropic receptor NMDA type subunit 2B—relief of magnesium ion blockade, and calcium overload, which activate downstream signaling pathways such as protein kinase C and calcium/calmodulin‐dependent protein kinase II. This process promotes the remodeling and membrane trafficking of α‐amino‐3‐hydroxy‐5‐methyl‐4‐isoxazolepropionic acid receptor subunits, collectively enhancing synaptic transmission efficiency (Guerrero‐Toro et al. 2022; Xie et al. 2023; Tsai et al. 2025). Moreover, the synergistic efficacy of serotonin and norepinephrine reuptake inhibitors on pain and comorbid mood disorders provides pharmacological evidence for the pivotal role of the monoaminergic system in these interactive mechanisms (Phelps et al. 2021). The aforementioned molecular abnormalities lead to significant pathological consequences: monoaminergic dysregulation weakens descending pain inhibitory pathways, impairs cognitive regulation and emotional homeostasis, and affects synaptic plasticity and neurogenesis; enhanced glutamatergic excitability persistently amplifies pain signals, forming the pathological basis for hyperalgesia and tactile allodynia; excessive pain signals overactivate the hypothalamic–pituitary–adrenal (HPA) axis, resulting in sustained overproduction of glucocorticoids (Spinieli et al. 2022). Prolonged exposure to high concentrations of glucocorticoids exerts neurotoxic effects on the hippocampus and prefrontal cortex, impairing learning, memory, and executive functions. It also suppresses the monoaminergic system, exacerbating neurotransmitter imbalance, ultimately creating a vicious cycle of “pain—neurotransmitter dysregulation—CI.” (Saunders et al. 2022; Ma et al. 2023).

### 3.3 The Interactive Mechanisms at the Brain Network Level

The interaction mechanisms at the brain network level aim to elucidate the emergent effects of the brain across temporal scales, from microscopic to macroscopic, and their intrinsic associations with brain functions such as cognition, emotion, and pain perception, as well as brain disorders including NP and AD. Large‐scale functional networks are formed based on the temporal correlations of neural activity, and their dynamic coordination constitutes the neural basis of higher cognitive functions like attention, memory, and executive control. In recent years, research on the comorbidity mechanisms of NP and CI has shifted from local brain region analyzes to a whole‐brain network dynamic perspective. Evidence indicates that under comorbid conditions, abnormal reorganization occurs in the dynamic interactions among three core networks—the DMN, the SN, and the CEN—forming the key network mechanism underlying the synchronized deterioration of pain perception and cognitive control (Fauchon et al. 2022; Xu et al. 2022; Chu et al. 2025). In a healthy brain, the SN functions as a dynamic switching hub that monitors salient internal and external stimuli, directing cognitive resources to flexibly switch between the self‐referential DMN and the externally focused CEN (Molnar‐Szakacs and Uddin 2022). However, under sustained endogenous salient stimulation in NP, the SN becomes hyperactivated, impairing its gating function. This results in cognitive resources being persistently fixated on pain processing, making it difficult to shift attention to external tasks (Baliki et al. 2024). Meanwhile, the CEN exhibits insufficient, delayed, or fragmented activation during task execution, with its limited resources competitively usurped by the DMN and SN, thereby impairing attention, working memory, and executive functions (Maesawa et al. 2021). Neuroimaging further reveals pathological structural changes associated with comorbidity: patients with chronic pain exhibit widespread structural remodeling, including reduced gray matter volume or cortical thinning in key network nodes such as the hippocampus, prefrontal cortex, and anterior cingulate cortex. These structural alterations are significantly correlated with memory decline, executive dysfunction, and pain progression (Zhang et al. 2023; Chaudhry et al. 2025). In summary, the synergistic effects of chronic pain‐induced stress, neuroinflammation, and a neurotoxic environment construct a brain network disease model characterized by hyperactivity of the SN as the triggering factor, imbalance in the interactions among the three core networks, and impaired neural plasticity as the structural basis. This ultimately leads to a vicious pathological cycle of “pain amplification—network imbalance exacerbation—CI” (Khalid et al. 2023; Liu et al. 2024).

### 3.4 Genetic‐Driven Upstream Factors

The comorbidity of NP and CI is not a linear causal event but the outcome of long‐term dynamic interactions between genetic susceptibility and environmental exposures. Its upstream driving system spans multiple levels—genetic, molecular, systemic, and environmental—shaping the disease trajectory through dual pathways: epigenetic mechanisms systemically regulate gene expression, while behavioral choices and environmental exposures are constrained by structural factors. This regulation initiates prior to symptom onset, laying an evolutionary foundation for the comorbidity (Clauw et al. 2024; Jiang, Geha, et al. 2025). At the molecular level, the apolipoprotein E ε4 allele exacerbates neuroinflammation, impairs synaptic plasticity and repair capacity, and weakens the brain's compensatory reserve (Liu et al. 2023; Yu et al. 2025). The Val66Met polymorphism of the *BDNF* gene can inhibit BDNF secretion, triggering neuronal dysfunction and synaptic plasticity damage. This not only reduces learning and memory abilities but also weakens descending pain inhibitory pathways, making Met carriers more susceptible to hippocampus‐dependent CI under chronic pain (Mazzitelli et al. 2025; Lechowicz et al. 2025). On the basis of genetic background, modifiable environmental behavioral factors accelerate the progression of comorbidities: disrupted sleep architecture interferes with the homeostatic remodeling necessary for neural plasticity, exacerbating central sensitization and neuroinflammation. Polysomnography confirms sleep abnormalities in chronic pain patients, while cognitive behavioral therapy can simultaneously improve sleep, pain, and cognition (Kim et al. 2025). Physical inactivity downregulates BDNF expression, weakens endogenous pain inhibition, disrupts cerebrovascular regulation and brain metabolic homeostasis, thereby promoting chronic pain and accelerating cognitive decline (Chen, Fang, et al. 2024; Jiang et al. 2024). Conversely, regular exercise can reverse these abnormalities (Kallings et al. 2021). Chronic stress and social isolation persistently activate the HPA axis, triggering glucocorticoid‐induced neurotoxicity and promoting peripheral and central inflammation, thereby forming a vicious stress‐inflammation cycle (Al Omran et al. 2022; Caria and Dall'Ò 2022). In summary, genetic and environmental factors do not simply add up independently but intertwine into a complex interactive network that jointly drives neuroinflammation, HPA axis dysregulation, synaptic plasticity impairment, and downstream brain network abnormalities, ultimately resulting in a comorbid progression model where pain perception and cognitive decline exacerbate each other.

### 3.5 A Perspective Based on Bidirectional Communication in GBA

The GBA is a bidirectional signaling network intertwining neural, endocrine, immune, and metabolic pathways, dynamically coordinating the functional interactions between the gastrointestinal tract and the CNS (Qu et al. 2024). Persistent pain activates the HPA axis, leading to continuous release of glucocorticoids and excessive excitation of the sympathetic nervous system, which triggers widespread neuroendocrine and autonomic imbalance. This chronic stress disrupts gut motility, mucus secretion, and local blood flow, undermining the homeostasis of the microenvironment (Chen et al. 2021). Meanwhile, stress‐related hormonal and neurotransmitter changes selectively promote the proliferation of pathogenic bacteria while inhibiting the colonization of beneficial symbionts, leading to dysbiosis in the microbial community's structure and function (Magni et al. 2023). Chronic stress, together with inflammatory factors, downregulates the expression of intestinal epithelial tight junction proteins (such as occludin and claudins), compromising barrier integrity and increasing permeability. This facilitates the translocation of microbial‐associated molecular patterns like lipopolysaccharides (LPS) into the bloodstream, triggering low‐grade systemic inflammation. Peripheral inflammation transmits signals via vagal afferent fibers expressing IL‐1β receptors, which directly project to the nucleus tractus solitarius and higher brain regions, or enters the CNS through areas of the ventricular system with a weakened BBB. This process drives microglia to shift toward a pro‐inflammatory M1 phenotype, releasing IL‐1β, TNF‐α, and ROS (Zeng et al. 2023; Zhang et al. 2025). Dysbiosis further exacerbates pathological processes through metabolic pathways: the synthesis of anti‐inflammatory and neuroprotective short‐chain fatty acids (butyrate, propionate) decreases, while tryptophan metabolism shifts toward quinolinic acid production, generating neurotoxic substances that enhance glutamate excitotoxicity (Tanaka et al. 2021). The aforementioned molecular cascades aggregate into multilayered effects: at the central level, persistent activation of central microglia and neuroinflammation maintain central sensitization, impairing synaptic plasticity in the hippocampus and prefrontal cortex, thereby directly driving the onset and progression of CI (Kong et al. 2023). In the gut‐brain interaction, the vagus nerve serves as a critical hub, transmitting afferent gut signals and mediating efferent top‐down autonomic regulation to achieve functional integration (Kasarello et al. 2023). Research has demonstrated that specific probiotics, such as gut Roseburia, can activate the vagus nerve pathway, inhibit activity in pain‐related brain regions, and promote butyrate production, thereby exerting analgesic effects, regulating neuroinflammation, and improving learning and memory deficits. This provides potential targets for interventions aimed at regulating NP and CI based on the GBA (Sun et al. 2024; Jiang, Huang, et al. 2025).

NP and CI often occur together in clinical settings, and this phenomenon is not coincidental; it stems from a complex and intertwined network of shared pathological mechanisms. Interactions across these levels mutually reinforce each other via positive feedback loops, creating a vicious cycle that intensifies pain and CI. Clarifying this integrated pathological panorama holds significant theoretical value for deepening the understanding of comorbidities and guiding neuroimmune‐brain network–targeted interventions. Currently, pharmacological treatments for CI associated with NP remain limited in addressing brain health, which has led to a growing research focus on non‐pharmacological interventions centered on natural products and nutritional regulation. For example, curcumin can promote hippocampal neurogenesis and repair synaptic plasticity, thereby improving pain behaviors and memory deficits (Du et al. 2021). Naringin inhibits glial cell inflammation and enhances BDNF signaling, alleviating hyperalgesia and CI (Hashemi et al. 2025). Probiotics modulate the gut microbiota and metabolites, influence protein expression and transcriptional activity in brain regions, and exert synergistic effects on analgesia and cognitive improvement (Abazari‐Bozhgani et al. 2025). Sublingual administration of vitamin B12 has also been shown to significantly reduce patients' pain and anxiety scores in the short term (Gharibpoor et al. 2022). Notably, Vitamin D has transcended its traditional role in calcium and phosphorus metabolism regulation and has been redefined as a pleiotropic secosteroid. Its active form, 1,25‐dihydroxyvitamin D_3_ (1,25‐(OH)_2_D_3_), can cross the BBB to modulate CNS functions, exerting immunoregulatory, neuroprotective, and endocrine regulatory effects, which closely align with the key mechanisms of comorbid NP‐CI. The following text will focus on Vitamin D as a dietary nutritional intervention in comorbidity management, providing a theoretical basis for optimizing Vitamin D nutritional status and developing related health strategies.

## 4 Dietary Nutrition, Public Health, and Neuroprotective Roles of Vitamin D

### 4.1 Dietary Sources and Metabolism of Vitamin D

Vitamin D is a fat‐soluble vitamin supplied to the body mainly through two pathways: the first is endogenous synthesis, where 7‐dehydrocholesterol (7‐DHC) in the skin is converted into Vitamin D_3_ upon exposure to ultraviolet B (UVB) radiation with wavelengths between 290 and 315 nm. The second is exogenous dietary intake, which primarily exists in three forms: Vitamin D_2_ (ergocalciferol), Vitamin D_3_ (cholecalciferol), and 25‐hydroxyvitamin D_3_ (25(OH)D_3_). Among dietary sources, Vitamin D_3_ is the most common form, predominantly found in animal‐based foods such as fatty fish, egg yolks, liver, and some fortified products like milk, yogurt, and butter. Vitamin D_2_ mainly comes from plant‐based foods, including ultraviolet‐irradiated mushrooms and fortified cereals and soy milk. Meanwhile, 25(OH)D_3_ naturally occurs in animal foods like eggs, fish, and milk, and its levels can be significantly increased by adding Vitamin D_3_ to animal feed. Vitamin D, as a prohormone, regulates calcium and phosphate homeostasis through the classical metabolic pathway, while also exerting broad physiological effects in various tissues via non‐classical pathways. After entering the bloodstream, Vitamin D is first converted in the liver by cytochrome P450 enzymes (CYP2R1 and CYP27A) into 25‐hydroxyvitamin D (25(OH)D). The serum concentration of this metabolite is widely recognized as the gold standard for assessing the body's Vitamin D nutritional status. Subsequently, under the coordinated regulation of the kidneys and parathyroid hormone, 25(OH)D undergoes further hydroxylation to form the fully biologically active 1,25‐dihydroxyvitamin D (1,25(OH)_2_D, also known as calcitriol). Finally, Vitamin D binding protein (VDBP) transports the active metabolite to target organs, where it binds to the intracellular nuclear receptor—the Vitamin D receptor (VDR)—to initiate downstream gene transcription, thereby exerting its extensive physiological regulatory functions (Figure 2) (Ortiz‐Prado et al. 2025).

**FIGURE 2:**
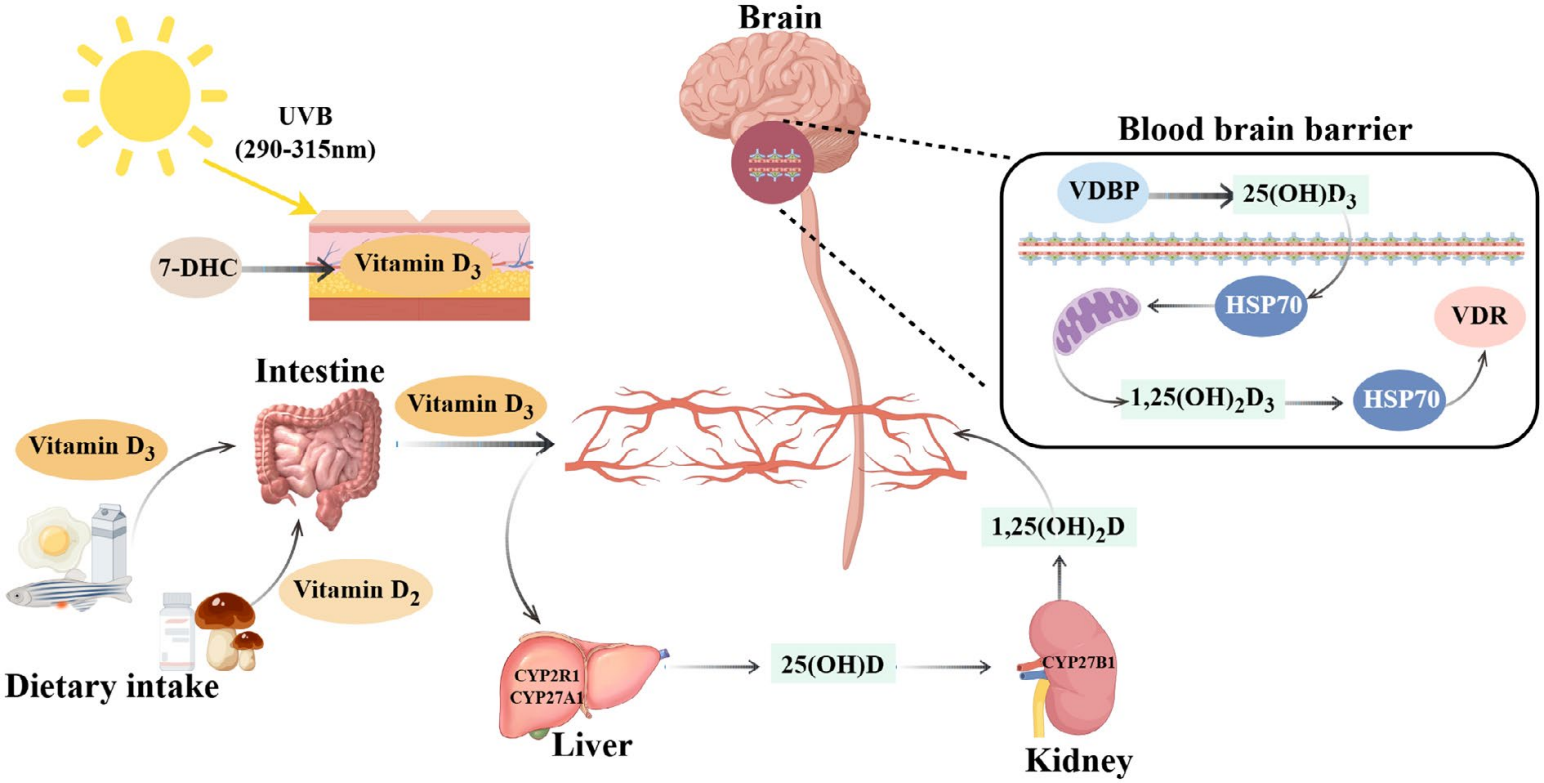
Sources and metabolism of Vitamin D. The main sources of vitamin D in the human body include dietary intake and synthesis in the skin through ultraviolet exposure. After vitamin D_3_ binds to vitamin D binding protein (VDBP), it is transported to the liver, where cytochrome P450 enzymes (CYP2R1 and CYP27A) catalyze its conversion to 25(OH)D. It then travels to the kidneys, where it is further hydroxylated under the regulation of parathyroid hormone to form the fully biologically active 1,25(OH)_2_D. Transported by VDBP to target tissues, it binds to the VDRto exert its wide‐ranging biological functions. The figure was created on https://www.Figdraw.com, has been authorized for use (copyright code: PRWAYdcd46). 1,25(OH)_2_D, 1,25‐dihydroxyvitamin D; 25(OH)D, 25‐hydroxyvitamin D; 25(OH)D_3_, 25‐hydroxyvitamin D_3_; 7‐DHC, 7‐dehydrocholesterol; HSP70, Heat Shock Protein 70; UVB, Ultraviolet B; VDBP, Vitamin D binding protein; VDR, Vitamin D receptor; Vitamin D_2_, Ergocalciferol; Vitamin D_3_, Cholecalciferol.

### 4.2 High‐Risk Susceptible Populations for VDD

The risk of VDD is not evenly distributed but is highly concentrated among high‐risk populations characterized by specific physiological, genetic, environmental, and behavioral factors. Systematic identification of these groups is a key prerequisite for developing precise health education strategies and individualized supplementation plans. Across the life cycle, infants experience a surge in skeletal mineralization demands, while breast milk contains very low levels of Vitamin D, making exclusively breastfed infants particularly vulnerable (Tan et al. 2020). Pregnant and lactating women face dual nutritional burdens since fetal bone development relies entirely on maternal supply; deficiency in these groups can lead not only to congenital rickets but also increases the risk of pregnancy complications (Durá‐Travé and Gallinas‐Victoriano 2023). Older adults typically suffer from multifactorial physiological deficiency due to reduced skin synthesis, decreased renal 1α‐hydroxylase activity hindering activation, combined with insufficient outdoor sunlight exposure and decreased dietary intake (Wu et al. 2025). Skin color and body weight are also independent and significant influencing factors. Individuals with darker skin have higher epidermal melanin content, which competitively absorbs UVB photons alongside 7‐DHC, resulting in a significantly reduced cutaneous synthesis rate under the same sunlight exposure. To achieve equivalent serum 25(OH)D levels, they may require 3 to 6 times longer UVB exposure compared to those with lighter skin, thereby markedly increasing their risk of deficiency (Clemens et al. 1982). Obese individuals experience a decrease in circulating bioavailability of Vitamin D because the fat‐soluble vitamin is extensively stored in white adipose tissue. Additionally, obesity is often accompanied by chronic low‐grade inflammation, which can disrupt Vitamin D metabolic pathways by downregulating the enzyme activities of hepatic CYP2R1 and renal CYP27B1. This inhibition reduces the production of 25(OH)D and its active form 1,25(OH)_2_D, further exacerbating the deficiency (Jirků et al. 2026). Lifestyle and environmental factors should not be overlooked either. Individuals who work indoors for extended periods, practice strict sun protection, or live in high‐latitude regions north of 35° N often have impaired cutaneous synthesis of Vitamin D—which accounts for 80% to 90% of the body's Vitamin D supply—or experience UVB radiation levels below the effective synthesis threshold during winter, resulting in generally low serum 25(OH)D levels (Alshamsi et al. 2025). Furthermore, pathological and iatrogenic risks represent another critical dimension. Patients with malabsorption syndromes—such as Crohn's disease, celiac disease, or post‐gastric bypass surgery—experience impaired absorption of fat‐soluble Vitamin D due to bile salt metabolism disorders or intestinal mucosal damage (Infantino et al. 2022). In patients with chronic liver disease, the activity of hepatic 25‐hydroxylase is downregulated, leading to impaired activation of Vitamin D and reduced production of 25(OH)D. Clinical studies have confirmed that VDD is common throughout the progression of chronic liver disease and that its severity positively correlates with disease advancement (Munoli et al. 2024). In patients with chronic kidney disease, once they reach stage 3, there is significant loss of functional renal parenchyma, accompanied by a progressive decline in CYP27B1 expression and activity (Helvig et al. 2010). Additionally, persistent phosphate retention and alterations in fibroblast growth factor‐23 levels during disease progression further feedback to inhibit CYP27B1 activity. The combined effect of these factors ultimately leads to impaired synthesis of the active form, 1,25(OH)_2_D (Nakatani et al. 2022). Additionally, individuals on long‐term anticonvulsants, glucocorticoids, and similar medications may develop iatrogenic VDD due to drug‐induced overexpression of hepatic microsomal cytochrome P450 enzymes, which accelerates the breakdown and inactivation of Vitamin D (Cheng, Zuo, et al. 2024). In summary, the distribution of high‐risk groups is characterized by a complex interplay of multiple dimensions and mechanisms. Only through systematic identification and stratified assessment of the aforementioned risk factors can early warning and personalized intervention for VDD be effectively achieved.

### 4.3 Vitamin D Supply Through the Synergy of Food Fortification Strategies and Dietary Patterns

Approximately half of the global population suffers from VDD, which has become a public health issue that cannot be ignored (Dunlop et al. 2025; Hamza et al. 2026). Prolonged indoor activities and insufficient effective sunlight exposure make endogenous synthesis inadequate to meet physiological needs, thereby increasing the importance of dietary intake. However, the variety of foods naturally rich in Vitamin D is limited, and a regular diet often fails to maintain sufficient levels. Therefore, it is imperative to enhance Vitamin D nutritional status in the population through a combined approach of food fortification and optimization of dietary patterns. Dietary diversification is the most fundamental and accessible means of nutrient supply. For populations primarily consuming plant‐based diets, such as vegetarians, and those relying on cereals as staple foods, precise adaptation tailored to their specific dietary patterns is necessary. Respecting long‐term eating habits enhances the acceptability and effectiveness of interventions. Building on this, mandatory food fortification promoted by national or regional authorities—due to its wide coverage and high compliance—can achieve uniform fortification in widely consumed staples like milk and cereals, forming the cornerstone of public nutrition interventions. Complementing this, voluntary fortification targets specific groups such as pregnant women and the elderly, implemented flexibly by manufacturers based on market demand, thereby creating a multi‐tiered complementary framework alongside mandatory policies. Traditional dietary supplementation and fortification have achieved certain successes; however, they still face limitations in accessibility, compliance, and long‐term sustainability. The recently emerging biofortification approach—through production technologies such as feed modification, ultraviolet irradiation, and genetic breeding—endogenously enhances the Vitamin D content in foods. This provides a practical supplementation pathway for rural and low‐resource populations, effectively addressing the shortcomings of traditional methods in terms of coverage depth. Currently, Vitamin D supply still faces multiple challenges, including insufficient public awareness, inadequate targeting of dietary interventions, limited policy universality, and low fortification coverage. Grounded in regional dietary patterns, economic development levels, and population risk differences, integrating fortification systems into sustainable food frameworks and implementing precise, differentiated interventions remain the most cost‐effective and systematic approaches to improving global Vitamin D nutritional status. In the future, policy guidance and interdisciplinary collaboration should be strengthened to facilitate the translation of related strategies from theoretical consensus to practical application, thereby truly benefiting diverse populations.

### 4.4 Global Public Health Challenges of VDD and Tertiary Prevention Strategies

Although the physiological functions of vitamin D are well established, its deficiency remains widespread globally. It is estimated that worldwide, 15.7% and 47.9% of participants have serum 25(OH)D levels below 30 nmol/L and 50 nmol/L, respectively, with particularly higher prevalence observed in populations from high‐latitude regions, during winter and spring seasons, and in low‐ to middle‐income countries (Cui et al. 2023). The risk of this deficiency is not evenly distributed; rather, it is primarily concentrated among populations with specific physiological characteristics, genetic backgrounds, environmental factors, and behavioral habits (Amrein et al. 2020). To address this global public health issue, there is an urgent need to develop evidence‐based and feasible public health strategies and standardized screening guidelines, which are crucial for the prevention and treatment of related diseases and for alleviating healthcare burdens (Cui et al. 2023). Public health strategies should prioritize population‐based preventive measures, combining precise risk stratification with targeted management of high‐risk groups, adhering to the principles of tertiary prevention, and establishing a comprehensive defense system that covers the entire spectrum of disease from prevention to treatment. Primary prevention focuses on universal prevention and health education, aiming to improve the overall nutritional status of the population and prevent nutrient deficiencies. Key measures include multi‐level comprehensive interventions: in areas with insufficient sunlight, mandatory Vitamin D fortification of widely consumed foods such as milk, plant‐based milk alternatives, and margarine is one of the most cost‐effective public health strategies. Additionally, the consumption of natural foods rich in Vitamin D (such as fatty fish, egg yolks, and animal liver) should be encouraged, along with the use of supplements to meet daily recommended intake levels. Public education should encompass safe sun exposure guidelines and the identification of risk factors to enhance public and individual awareness of risks, ultimately improving the overall nutritional status of the population (Demay et al. 2024; Wu et al. 2025). Secondary prevention focuses on early screening and intervention, emphasizing the identification and intervention of high‐risk individuals before clinical symptoms appear, thereby interrupting disease progression. This strategy primarily targets individuals with bone diseases, metabolic‐related conditions, and specific physiological states such as obesity, the elderly, and pregnant women, using standardized serum 25(OH)D testing for objective assessment. Although there are slight differences in the specific threshold values set by various authoritative bodies, a core diagnostic standard has been largely unified (Cappola et al. 2023; Giustina et al. 2024; CNS‐HM). Table 1 details the classification standards, corresponding levels, and clinical significance based on serum 25(OH)D concentrations from different authorities, aiming to provide a unified diagnostic basis for inter‐laboratory testing and clinical research, ensuring consistency and accuracy in result interpretation. Tertiary prevention centers on disease management and treatment, targeting patients diagnosed with VDD and related diseases. It involves standardized treatment (such as adequate doses of Vitamin D_3_ supplementation) and multidisciplinary collaborative management to improve clinical outcomes, prevent recurrence, and reduce the risk of complications. In summary, public health strategies for Vitamin D should prioritize population‐based prevention and use screening as a precise intervention tool for high‐risk groups. Future research needs to further elucidate the mechanisms of Vitamin D in diseases beyond bone health, clarify its optimal serum concentration thresholds, optimize food fortification systems, and develop convenient testing technologies. These efforts aim to enhance the scientific basis of public health decision‐making and improve the precision of individualized health management.

**TABLE 1:** Reference levels of serum 25(OH)D according to the latest guidelines (2023–2025).

Status classification	Serum 25 (OH) D content				Clinical significance
	ES	ESE	IOF	CNS	
Severe deficiency	≤ 12 ng/mL (≤ 30 nmol/L)	≤ 12 ng/mL (≤ 30 nmol/L)	< 12 ng/mL (< 30 nmol/L)	< 12 ng/mL (< 30 nmol/L)	Directly associated with rickets and osteomalacia, it is a category that clearly requires drug treatment
Deficiency	Not separately defined	Not separately defined	< 20 ng/mL (< 50 nmol/L)	12–< 20 ng/mL (30–< 50 nmol/L) (classified as “insufficiency”)	Insufficient maintenance of bone and muscle health is associated with secondary hyperthyroidism, decreased bone density, and increased risk of fractures
Insufficiency	Category withdrawn (20–30 ng/mL no longer actionable)	Category withdrawn (same as ES 2024)	20–< 30 ng/mL (50—< 75 nmol/L)		For high‐risk populations (e.g., elderly, osteoporosis patients), this level may be insufficient to optimize calcium absorption and muscle function. It is recommended to increase the level to a sufficient range through supplements
Adequate/Normal	No sufficiency threshold set (≥ 30 ng/mL target withdrawn)	General population: no threshold set; HypoPT patients: ≥ 30 ng/mL (≥ 75 nmol/L)	Website 2026: ≥ 20 ng/mL (≥ 50 nmol/L); 2010 statement: ≥ 30 ng/mL (≥ 75 nmol/L)	≥ 20 ng/mL (≥ 50 nmol/L)	Consensus target range ensuring sufficient body reserve to support bone health and meet physiological needs in the absence of sunlight
Potential poisoning/Upper limit of concern	> 50 ng/mL (> 125 nmol/L)	> 50 ng/mL (> 125 nmol/L)	> 150 ng/mL (> 375 nmol/L)	> 224 ng/mL (> 560 nmol/L)	Poisoning occurs only at extremely high serum concentrations, usually due to long‐term inappropriate high‐dose supplementation

Abbreviations: CNS, Chinese Nutrition Society; ES, Endocrine Society; ESE, European Society of Endocrinology; IOF, International Osteoporosis Foundation.

### 4.5 Distribution of VDR in the CNS and Its Neuroprotective Effects

Vitamin D, beyond regulating bone metabolism, is also involved in the pathological processes of cardiovascular diseases, metabolism, hypertension, and cancer. In 1991, Lemire and colleagues first demonstrated in an autoimmune encephalomyelitis model that Vitamin D is a key factor for myelin regeneration, marking the beginning of its neurobiological research (Lemire and Archer 1991). Subsequent studies have shown that Vitamin D and its receptor can delay neurodegenerative changes by promoting the synthesis of neurotrophic factors, regulating neurotransmitter metabolism, and maintaining the integrity of the BBB through immune modulation, anti‐inflammatory, and antioxidant effects (Pietruszkiewicz et al. 2025). Deficiency of Vitamin D can lead to dysfunction of the GABAergic interneuron network, manifested as increased pain sensitivity and reduced cognitive flexibility (Kasatkina et al. 2020; Zhang et al. 2024). VDR exhibits region‐specific distribution in the CNS, forming the structural basis for its diverse neural functions. It is highly expressed in the hippocampal CA1–CA4 regions and dentate gyrus cells; the latter enhances synaptic plasticity, counteracts excitotoxicity and oxidative stress, and participates in memory consolidation and neuroprotection (Langub et al. 2001; Walbert et al. 2001). VDR in the amygdala and nucleus accumbens may regulate emotion and reward‐related behaviors (Liu et al. 2021). In the hypothalamus, VDR is densely distributed in the suprachiasmatic nucleus (SCN) and paraventricular nucleus (PVN), where it can bind to Vitamin D response elements in the arginine vasopressin (AVP) gene and regulate stress, social behavior, and pain through the AVP and oxytocin systems (Ye et al. 2023). Within the SCN, VDR is primarily located in vasoactive intestinal peptide and AVP neurons, where it influences the expression of *BMAL1, Per2*, and *BDNF*, maintaining circadian rhythm stability. This corresponds with the sleep disturbances observed in individuals with VDD (Abboud 2022; Cai et al. 2025). In the arcuate nucleus, Vitamin D may be involved in energy balance and mitigate damage caused by a high‐fat diet. UVB irradiation can activate the pro‐opiomelanocortin system, upregulating α‐melanocyte‐stimulating hormone and melanocortin 4 receptor, while downregulating Agouti‐related peptide, thereby exerting an appetite‐suppressing effect. Additionally, VDS can alleviate inflammation and oxidative stress in the hypothalamus and hippocampus of obese rats by modulating the NF‐κB pathway and acetylcholine levels, leading to improved cognition (Skobowiat and Slominski 2016; Farhangi et al. 2017). Dopaminergic neurons in the substantia nigra pars compacta also highly express VDR; its activation can inhibit microglial activation, enhance antioxidant defenses, and promote the release of glial cell‐derived neurotrophic factor (Pertile et al. 2018). VDR can directly regulate the transcription of tryptophan hydroxylase (TPH), affecting serotonergic transmission and participating in the habenula's regulation of emotion, anxiety, and sleep, providing a molecular basis for Vitamin D's adjunctive treatment in mood disorders (Kaneko et al. 2015). In summary, the specific distribution of VDR in key brain regions provides the structural and functional foundation for Vitamin D's involvement in cognition, emotion, circadian rhythm, metabolism, and motor control.

## 5 Mechanistic Basis of Vitamin D Application in NP and CI

Vitamin D inhibits neuroinflammatory cascades through multi‐target mechanisms, alleviates oxidative stress‐induced neuronal damage, and upregulates neurotrophic signaling pathways, thereby maintaining CNS homeostasis, preserving brain functional integrity, and delaying neurodegenerative progression. VDD has been identified as a shared risk factor for NP and CI; thus, supplementation can simultaneously intervene in the molecular pathways common to both conditions at multiple pathological levels, exerting a synergistic therapeutic effect (Figure 3).

**FIGURE 3:**
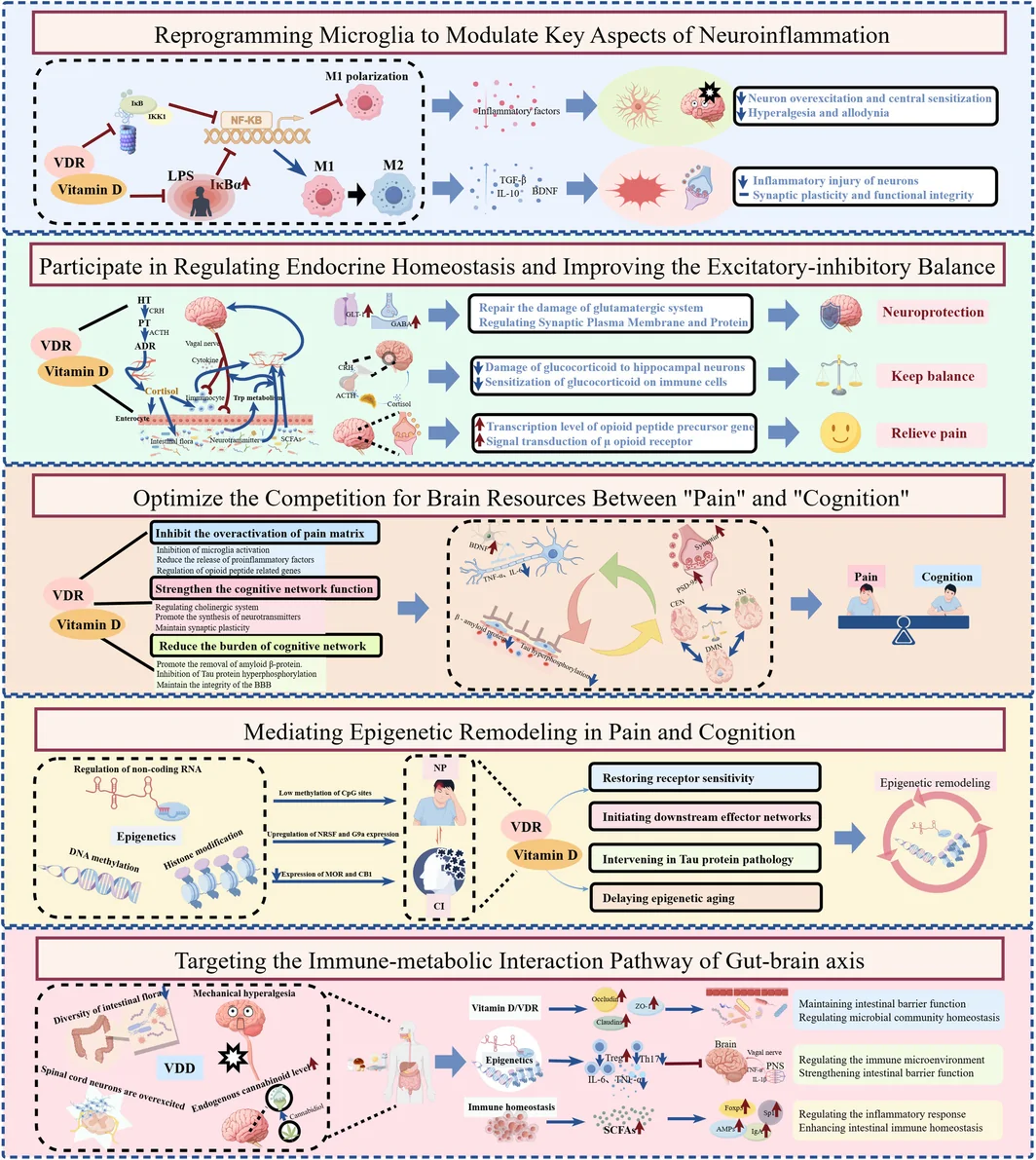
Multiple protective mechanisms of Vitamin D in NP‐CI comorbidity. Vitamin D can participate in regulating the pathophysiological processes of comorbidity between NP and CI through multiple targets, including inhibiting the cascade of neuroinflammation, maintaining neuroendocrine homeostasis, optimizing brain resource competition, mediating epigenetic remodeling, and targeting the immune‐metabolic pathways of the gut‐brain axis. The figure was created on https://www.Figdraw.com, has been authorized for use (copyright code: IAPII96926). ACTH, Adrenocorticotropic hormone; ADR, Adrenal; BBB, Blood–brain barrier; BDNF, Brain‐derived neurotrophic factor; CB1, Cannabinoid receptor 1; CEN, Central executive network; CI, Cognitive impairment; CpG sites, Cytosine‐phosphate‐Guanine site; CRH, Corticotropin‐releasing hormone; DMN, Default mode network; HT, Hypothalamus; LPS, Lipopolysaccharides; MOR, μ‐opioid receptor; NP, Neuropathic pain; NRSF, Neuron‐restrictive silencer factor; PNS, Peripheral nervous system; PT, Pituitary; SCFAs, Short‐chain fatty acids; SN, Salience network; VDD, Vitamin D deficiency; VDR, Vitamin D receptor.

### 5.1 Reprogramming Microglia to Modulate Key Aspects of Neuroinflammation

The excessive activation of microglia in key regions of the spinal cord and brain, along with the polarization of the pro‐inflammatory M1 phenotype, is an important driving factor in the development of comorbidity between NP and CI. M1‐type microglia release large amounts of pro‐inflammatory cytokines such as IL‐1β, TNF‐α, and IL‐6, which, through central sensitization and synaptic damage, directly induce abnormal pain perception and cognitive decline. Vitamin D plays a crucial role in regulating neuroimmune function, particularly demonstrating clear potential by mediating microglial phenotype switching (Mirarchi et al. 2023). The active form 1,25(OH)_2_D binds to the VDR to form a transcriptional complex that inhibits the NF‐κB pathway through a dual mechanism: on one hand, VDR physically interacts with IκB kinase β, attenuating TNF‐α‐induced p65 nuclear translocation and NF‐κB activity (Chen et al. 2013). On the other hand, it induces VDR expression and promotes its interaction with the p50 subunit of NF‐κB, thereby weakening the binding between KLF5 and the NF‐κB p50 subunit, ultimately suppressing inflammation triggered by pathogen‐associated molecular patterns such as LPS. Based on this regulatory network, Vitamin D not only inhibits microglial polarization toward the pro‐inflammatory M1 phenotype but also actively promotes their shift to the anti‐inflammatory, reparative M2 phenotype. This facilitates the release of protective factors such as IL‐10, TGF‐β, and BDNF, alleviating neuronal inflammatory damage and preserving synaptic plasticity and structural integrity (Calvello et al. 2017).

### 5.2 Participate in Regulating Endocrine Homeostasis and Improving the Excitatory‐Inhibitory Balance

Vitamin D, through its nuclear receptor widely distributed and activated in the nervous and endocrine systems, provides a molecular basis for regulating the local neural microenvironment and maintaining homeostasis of the stress response system. At the neurotransmitter level, Vitamin D bidirectionally modulates the HPA axis function and central neurotransmitter activity by regulating the expression of TPH 2, serotonin transporter, and monoamine oxidase A. This influences the homeostasis of the 5‐HT system, thereby participating in the regulation of cognitive processes such as executive function and sensory gating (Bostan et al. 2025). Additionally, Vitamin D can improve behavioral outcomes in Parkinson's disease mouse models through the VDR/poly (ADP‐ribose) polymerase‐1 (PARP1) signaling pathway, restoring dopaminergic neuron damage and reducing the activation of PARP1‐related signaling pathways (Hu et al. 2021). Simultaneously, it reduces β‐amyloid deposition and inhibits abnormal proliferation of GABAergic astrocytes, thereby correcting GABAergic dysfunction, alleviating neuroinflammation, and improving cognitive performance (Kang et al. 2024). At the synaptic level, Vitamin D directly regulates the excitatory‐inhibitory balance by upregulating the expression of the astrocytic glutamate transporter GLT‐1, accelerating glutamate clearance from the synaptic cleft to limit excitotoxicity (Beaudin and Welsh 2016). Simultaneously, it promotes GABA synthesis and modulates GABA receptor function, enhancing central inhibitory tone and preserving neuronal structural integrity (Kasatkina et al. 2020). At the systemic homeostasis level, Vitamin D exerts neuroprotective effects by correcting dysregulation of the HPA axis negative feedback. NP, as a persistent stressor, activates the HPA axis and elevates glucocorticoid levels (Corriger et al. 2019). VDS, on one hand, upregulates the expression and function of hippocampal glucocorticoid receptors, strengthening their mediated negative feedback regulation (Camargo et al. 2020); on the other hand, it directly suppresses the transcription of corticotropin‐releasing hormone in the hypothalamic PVN via VDR, thereby attenuating the overactivation signals of the HPA axis (Su et al. 2021). Moreover, VDD significantly impairs opioid peptide signaling in the brainstem and spinal cord, reducing the body's ability to counteract nociceptive stimuli and leading to lowered pain thresholds (Zeng et al. 2022). Conversely, VDS not only modulates the expression of genes related to the opioid signaling pathway, including Penk and Pomc, but also functionally interacts with the μ‐opioid receptor system, providing a theoretical basis for its analgesic mechanism independent of anti‐inflammatory effects (Poisbeau et al. 2019; Kemény et al. 2021).

### 5.3 Optimize the Competition for Brain Resources Between “Pain” and “Cognition”

Pain not only arises from localized sensory processing abnormalities but is also closely associated with the widely distributed “pain matrix” (Yao, Chen, and Chen 2023). Notably, these brain regions significantly overlap spatially and continuously interact functionally with networks supporting higher cognitive functions, including the DMN, SN, and CEN (Zheng, Ruan, et al. 2024). The chronic persistence of pain can induce CI by occupying or disrupting neural resources shared among these networks. The regulatory role of Vitamin D in this resource competition relies on the widespread distribution of its receptor and metabolic enzymes within the CNS and is mediated through multiple pathways such as neuroprotection, anti‐inflammation, and synaptic plasticity modulation (De Ridder et al. 2022). On one hand, Vitamin D inhibits the hyperactivation of the pain matrix, thereby reducing competition for cognitive resources: it suppresses microglial activation and the release of pro‐inflammatory cytokines, diminishes abnormal excitation within pain pathways, promotes neuronal repair, and lessens the overall consumption of cognitive resources by the pain matrix (Alessio et al. 2021). Simultaneously, Vitamin D regulates the expression of endogenous opioid‐related genes (*POMC, PDYN, PENK*), enhancing central analgesic effects and thereby decreasing the pain matrix's consumption of neural resources at the source (Poisbeau et al. 2019). On the other hand, Vitamin D directly enhances cognitive network function and improves resource competition patterns. In key brain regions such as the hippocampus and prefrontal cortex, it activates choline acetyltransferase to regulate the cholinergic system, upregulates TPH activity to promote 5‐HT synthesis, and increases neurotransmitter transmission efficiency (Alrefaie and Alhayani 2015; Lucas et al. 2018). At the same time, it modulates neurotrophic factor levels, promotes the expression of synaptic proteins (synaptophysin, PSD‐95), maintains synaptic plasticity, and strengthens the cognitive network's ability to acquire and utilize neural resources (Kouba et al. 2023; Kouba and Rodrigues 2024). Moreover, VDD not only disrupts local neural synchronization but also progressively impairs overall network connectivity, thereby affecting cognitive function, emotional regulation, and social abilities. Studies have confirmed that the role of Vitamin D extends beyond specific brain regions, aiding in the coordination and maintenance of information communication between different brain areas, optimizing the stability and operational efficiency of neural networks (Tian et al. 2025). In summary, through bidirectional synergistic regulation of the pain perception system and higher cognitive systems, Vitamin D holds promise in breaking the vicious cycle driven by the imbalance of brain network resource competition between pain and cognitive decline.

### 5.4 Mediating Epigenetic Remodeling in Pain and Cognition

Epigenetic regulation serves as a crucial link between environmental stimuli and genomic transcriptional reprogramming. It precisely modulates gene expression through mechanisms such as histone modification and DNA methylation, directly contributing to the maintenance of neuronal structural plasticity and dynamic synaptic function. Moreover, it is closely associated with the onset and progression of abnormal pain perception and CI (Singh and Paramanik 2024; Idahor et al. 2025). Quantitative analysis of whole‐genome methylation revealed hypomethylation at CpG sites in the dorsal root ganglia of the spinal nerve ligation model (Garriga et al. 2018). This was accompanied by upregulated expression of neuron‐restrictive silencer factor and the histone methyltransferase G9a in the dorsal root ganglia (Luo et al. 2020; Su et al. 2022). The latter catalyzes an increase in H3K9me2 levels, repressing the transcription of μ‐opioid receptor and cannabinoid receptor 1, thereby weakening the analgesic efficacy of opioids (Zhang et al. 2016). However, G9a inhibitors or gene silencing can reverse these phenotypes (Li and Tao 2022). Strath and colleagues also observed significant differences in DNA methylation of the CYP27B1 gene, which encodes a key enzyme in vitamin D metabolism, between pain and non‐pain populations. Additionally, the promoter region of the VDR gene exhibited a hypermethylated state (Strath et al. 2023). Based on this, the role of vitamin D in epigenetic regulation of pain and cognition has attracted scholarly attention. Current research mainly focuses on histone modifications (especially acetylation) and DNA methylation. Studies suggest that vitamin D may improve pain and CI through epigenetic mechanisms by regulating the following pathway: (1) Restoring receptor sensitivity. Under pain conditions, the promoter region of the VDR gene becomes hypermethylated. Vitamin D can reverse this epigenetic modification to restore normal VDR expression, thereby enhancing the responsiveness of neurons and glial cells to ligands (Fetahu et al. 2014; Zainuddin et al. 2025). (2) Initiating downstream effector networks. Activated VDR regulates target genes associated with inflammatory responses, neural plasticity, and synaptic function, while also modulating the methylation levels of metabolic enzymes CYP27B1 and CYP24A1. This helps maintain central vitamin D homeostasis, providing a metabolic foundation for signal transduction (Wjst et al. 2010). (3) Intervening in Tau protein pathology. Vitamin D upregulates the expression of methylenetetrahydrofolate reductase and leucine carboxyl methyltransferase‐1, enhancing the methylation activity of protein phosphatase 2A. This reduces the aggregation of hyperphosphorylated Tau protein, thereby directly protecting cognitive function (Pan et al. 2021). (4) Delaying epigenetic aging. By slowing down the acceleration of the epigenetic clock, it indirectly mitigates age‐related pain and cognitive decline (Strath et al. 2022; Minami et al. 2025).

### 5.5 Targeting the Immune‐Metabolic Interaction Pathway of GBA

The association between gut microbiota and CNS disorders has become a research hotspot. Existing evidence indicates that the microbiota, along with its metabolites and immune mediators, interact with neural structures to form a multi‐level network connecting the gut microbiota, PNS, and CNS, collectively mediating the onset and progression of pain and CI (Santos et al. 2024). Vitamin D, as a crucial signaling molecule, has garnered significant attention in brain health interventions for anxiety, depression, and aging‐related CNS diseases due to its multifaceted roles in regulating intestinal barrier function, immune homeostasis, and microbiota metabolism (Renteria et al. 2024; Ullah 2025). Animal studies have shown that VDD leads to reduced microbial diversity, altered pain perception, and changes in neuronal excitability; supplementation with Vitamin D, through the VDR signaling pathway, regulates tight junction proteins (occludin, ZO‐1, and claudin‐2), enhances mucosal barrier integrity, reverses dysbiosis, and promotes microbial diversity (Guida et al. 2020; Sun and Zhang 2022). Meanwhile, Vitamin D systemically reduces levels of pro‐inflammatory factors such as IL‐6 and TNF‐α by inhibiting Th1 and Th17 cells and promoting the differentiation of regulatory T cells. This immunomodulatory effect blocks the transmission of peripheral inflammation to the CNS from the immune microenvironment perspective, synergizing with barrier reinforcement to jointly maintain GBA homeostasis (Fakhoury et al. 2020; Hwang et al. 2023). Vitamin D also participates in the aforementioned regulation by modulating short‐chain fatty acids (SCFAs) produced through gut microbiota metabolism (Vinogradova et al. 2025). Among these, butyrate enhances acetylation of the VDR promoter region and upregulates VDR expression by inhibiting histone deacetylase 3 (HDAC3). This effect synergizes with 1,25‐(OH)_2_D_3_ to activate both the HDAC3‐VDR and aryl hydrocarbon receptor‐VDR axes, driving IL‐22‐mediated tissue repair, antimicrobial peptide production, autophagy, and tight junction restoration, thereby enhancing anti‐inflammatory responses through multiple pathways (Liao et al. 2025; Huang 2026). Additionally, VDR expressed in the intestinal barrier can induce the production of antimicrobial peptides, which enhance innate gut immunity by disrupting the integrity of pathogen cell membranes (Tabassum et al. 2023). Notably, SCFAs can also upregulate Vitamin D expression by enhancing the activity of the transcription factor Sp1, forming a positive feedback loop that further stabilizes GBA homeostasis (Moreno‐Torres et al. 2022).

In summary, Vitamin D establishes a comprehensive theoretical intervention framework for the comorbidity of NP and CI through a multi‐target synergistic mechanism. The core logic lies in that Vitamin D, rather than acting via a single pathway, mediates transcriptional regulation through the VDR receptor, exerting systemic effects across five dimensions: (1) neuroimmunity—suppressing M1 polarization, promoting M2 transformation, and inhibiting the NF‐κB pathway; (2) neuroendocrine regulation—modulating the HPA axis, 5‐HT, and GABA systems to restore excitatory‐inhibitory balance; (3) brain network resource competition—inhibiting overactivation of the pain matrix while strengthening functional connectivity between the DMN and executive networks; (4) epigenetic remodeling—reversing VDR promoter methylation, regulating histone modifications, restoring receptor sensitivity, and inhibiting Tau protein pathology; (5) GBA interaction—maintaining intestinal barrier integrity, regulating microbiota‐derived metabolites such as SCFAs, and reinforcing the HDAC3‐VDR positive feedback loop. Therefore, this mechanism establishes a closed‐loop regulatory system extending from peripheral immune metabolism to CNS homeostasis. By interrupting the vicious cycle between pain and CI, it offers integrated therapeutic targets encompassing anti‐inflammatory, analgesic, neurotrophic, and synaptic protective effects for clinical comorbidity interventions. Subsequent sections will systematically review foundational research and clinical evidence, integrate cutting‐edge advancements, and further elucidate the potential application of Vitamin D in the comprehensive treatment of comorbid NP and CI.

## 6 Clinical Potential and Limitations of Vitamin D in the Comorbidity of NP and CI

Current clinical treatment for NP primarily relies on opioids and non‐steroidal anti‐inflammatory drugs. However, the long‐term use of these medications not only poses a risk of addiction but may also increase the risk of CI. In recent years, nutritional interventions have garnered widespread attention in the prevention and treatment of chronic diseases due to their multi‐target and low‐risk systemic regulatory characteristics. The key mechanism lies in correcting nutritional deficiencies and restoring normal cellular and tissue metabolism, thereby intervening in disease progression from a pathophysiological perspective. In the preceding text, we discussed the potential synergistic regulatory effects of Vitamin D on pain and cognition through multiple mechanisms. However, current clinical intervention evidence for Vitamin D in NP and CI still faces significant heterogeneity regarding study populations, baseline Vitamin D levels, supplementation protocols, and outcome measures. Moreover, high‐quality RCTs targeting patients with comorbid NP and CI, with both pain and cognitive improvement as primary endpoints, are still lacking. Therefore, the efficacy of Vitamin D in this comorbid condition remains to be further validated.

### 6.1 A Review of Clinical Evidence for Vitamin D in NP

With the advancement of research, the role of vitamin D in pain perception and regulation has gradually gained attention. Numerous observational studies suggest that lower vitamin D levels are negatively correlated with the prevalence and severity of various NP, such as diabetic peripheral neuropathy (DPN), spinal cord injury, and postherpetic neuralgia (PHN) (Seetan et al. 2021; Shi, He, et al. 2025; Atoum et al. 2026). Based on its neuroprotective, immunomodulatory, anti‐inflammatory, and pain signaling regulatory properties, vitamin D is regarded as a potential adjunctive strategy for managing NP. Existing RCT evidence shows significant heterogeneity: most studies targeting specific populations confirm that vitamin D supplementation (VDS) has a positive effect on managing various types of NP and is generally safe; however, some large‐scale trials and systematic reviews have not observed consistent benefits. Table 2 summarizes recent evidence on the efficacy of vitamin D in different types of NP, covering key factors such as study design, dosing regimens, intervention duration, and outcome measures. Interventional studies have indeed partially confirmed the adjunct analgesic potential of vitamin D in NP, especially when participants have a clear VDD. A 2025 meta‐analysis including four RCTs with a total of 320 patients suffering from DPN showed that vitamin D significantly reduced pain in the short term compared to placebo (SMD = −0.85; 95% CI: −1.07 to −0.62), equivalent to an 11‐point decrease on commonly used pain scales. However, this analysis exhibited moderate heterogeneity (*I* ^2^ = 61.4%), a relatively small overall sample size, limited methodological quality, and lacked data on mid‐ to long‐term efficacy and safety outcomes (Gilbody and Gilbody 2025). There are significant differences in dosing regimens among studies. In the RCT conducted by Pinzon et al., daily supplementation of 2000 IU vitamin D as an adjunct therapy led to pain relief as well as improvements in sleep quality, daily functioning, and mood after 8 weeks. Similarly, Mehta S et al. administered 60,000 IU of vitamin D weekly and observed improvements in patients' NP and symptoms of foot nerve degeneration (Mehta et al. 2021; Pinzon and Angela 2025). For other causes of NP, such as carpal tunnel syndrome, tumor‐related pain, spinal stenosis, and endometriosis, despite heterogeneity in VDS doses and regimens, many clinical studies have still observed the potential applications of vitamin D. Notably, in 2025, Shi et al. employed mediation Mendelian randomization methods, using large‐scale genome‐wide association study data to genetically dissect the causal relationships among immune cells, serum micronutrients, and NP. This provided crucial causal evidence supporting the adjunctive use of vitamin D in NP management. The study confirmed that serum vitamin D levels play a key mediating role in the protective effects of specific immune cells against NP, revealing vitamin D as a central mediator linking immune dysregulation and pain occurrence. These findings offer mechanistic support for vitamin D's adjunctive role in NP treatment, moving beyond mere correlation (Shi, Yang, et al. 2025).

**TABLE 2:** Summary of studies on vitamin D intervention in pain (including NP and mixed pain).

First author (year)	Study type	Sample size	Patient type	Pain mechanism	Dose of vitamin D	Duration	Findings	Test used
Yang et al. (2026)	NR	745	DPN	NP	Single injection: 300,000–600,000 IU 2059–5000 IU per day 28,000–50,000 IU per week	Single injection, 8–12 weeks	Assessment and supplementation of Vitamin D can serve as an adjunctive strategy for managing painful DPN	VAS, MPQ, DN4, Neuro‐QoL, MNSI, NPS, NRS and BPI
Pinzon and Angela (2025)	RCT	60	PDN	NP	2000 IU per day	8 weeks	Reduce pain and improve sleep quality, daily functioning, and mood	VAS and BPI
Gilbody and Gilbody (2025)	Systematic review and meta‐analysis	320	PDN	NP	4000 IU per day–50,000 IU per week	8–12 weeks	Reduce short‐term pain in PDN	VAS, NSS and NPS
Mehta et al. (2021)	RCT	150	DPN	NP	60,000 IU per week	6 months	Improved symptoms of neuropathic pain and foot neurodegenerative injury	DN4, NPSI, and ITTT
Pinzon et al. (2021)	RCT	68	DPN	NP	5000 IU per day	8 weeks	VAS and burning pain scores decreased, and emotions also improved	VAS, NRS and BPI
Davoudi et al. (2021)	RCT	225	PDN	NP	4000 IU per day	12 weeks	Reduce pain severity and pain‐related disability, thereby improving patients' quality of life	PDI, NPS and Neuro‐QoL
Reda Halawa et al. (2021)	CC	178	PN	NP	200,000 IU per month	3 months	Improves glycemic parameters, PN score and severity	DN4 and SF‐MPQ
Savadjani et al. (2023)	SR	304	CTS	NP	Not reported	12–24 weeks	Functional score, nerve conduction, and pain, were improved	VAS, DASH and BCTQ
Noorizadeh et al. (2022)	RCT	105	CTS	NP	1000 IU per day/50,000 IU per week	6 months	Reduce the severity of pain, improve postoperative symptoms and functional impairments	BSFQ and VAS
Samant and Sane (2022)	POS	42	CTS	NP	60,000 IU per week	3 months	improvement of patients' function and symptoms, and relief of related pain	VAS and BCTQ
Anusitviwat et al. (2021)	SR	258	CTS	NP	1000 IU per day–60,000 IU per week	12–24 weeks	Pain relief, improvement of functional status, and enhancement of sensory nerve conduction velocity	VAS, functional score, muscle strength and nerve conduction velocity
Korkut et al. (2021)	POS	55	CTS	NP	Loading dose: 50,000 IU per week for 8 weeks Maintenance dose: 3000 to 6000 IU per day	3 months	Improvement of patients' symptoms, functional status, and electrophysiological findings	VAS and DASH
Almassri et al. (2025)	RCT	88	Breast cancer	Mixed Pain	600 mg ω3 per day +50,000 IU Vitamin D per week	9 weeks	Both patients' quality of life and blood inflammatory markers improved	Blood inflammatory markers, QLQ‐C30 and QLQ‐BR23 questionnaires
Hysa et al. (2025)	RLI	58	PMR	Mixed Pain	Average 2100 IU per day	3 months	Clinical symptoms of patients in remission	PMR‐AS and serum inflammatory biomarkers
Ko et al. (2023)	RCT	51	Lumbar spinal stenosis	Mixed Pain	Single injection: 200,000 IU 800 IU orally at 12 weeks	6 months	Improvement of lower back pain, as well as enhancement of spinal function and quality of life	VAS, ODI, RMDQ and SF‐36
Zarrati et al. (2022)	SR	564	Cancer‐related pain	Mixed Pain	2000–50,000 IU per day	12–52 weeks	Alleviate the related pain caused by cancer patients' treatment	HAQ‐II, BPI, FIQ and VAS
Mehdizadehkashi et al. (2021)	RCT	60	Endometriosis	Mixed Pain	50,000 IU every 2 weeks	12 weeks	Improvement of pelvic pain	Malondialdehyde, clinical symptoms and other metabolic profile
Ali et al. (2021)	QED	135	MSD	Mixed Pain	40,000 IU per week	8 weeks	More effective in relieving pain than simple physical therapy	BPI

Abbreviations: BCTQ, Boston carpal tunnel questionnaire; BPI, Brief pain inventory; BSFQ, Boston symptomatic and functional questionnaire; CC, Case–control study; CTS, Carpal tunnel syndrome; DASH, Disabilities of the arm, shoulder and hand; DN4, Douleur neuropathique 4; DPN, Diabetic peripheral neuropathy; FIQ, Fibromyalgia impact questionnaire; HAQ‐II, Health assessment questionnaire II; ITTT, Ipswich touch the toes test questionnaire; MNSI, Michigan neuropathy screening instrument; MPQ, McGill pain questionnaire; MSD, Musculoskeletal disorders; Neuro‐QoL, Quality of life in neurological disorders; NP, Neuropathic pain; NPS, Neuropathic pain scale; NPSI, Neuropathic pain symptom inventory; NR, Narrative review; NRS, Numerical rating scale; ODI, Oswestry disability index; PDI, Pain disability index; PDN, Painful diabetic neuropathy; PMR, Polymyalgia rheumatica; PN, Peripheral neuropathy; QLQ‐BR23, Quality of Life Questionnaire Breast Cancer Module 23; QLQ‐C30, Quality of Life Questionnaire Core 30; RCT, Randomized controlled trial; RMDQ, Roland–morris disability questionnaire; SF‐36, 36‐Item short form health survey; SF‐MPQ, Short‐form McGill pain questionnaire; SR, Systematic review; VAS, Visual analog scale.

From this series of studies, we can observe that positive results generally include susceptible populations with baseline VDD or high risk of deficiency. But does VDS provide similar benefits in the general population? Indeed, not all evidence points to positive conclusions. Especially among individuals with sufficient vitamin D levels, many supplementation trials have yielded negative results in pain patients. For instance, a large‐scale RCT pain analysis based on the D‐Health cohort showed that in a generally sufficient population with an average baseline serum 25(OH)D concentration above 50 nmol/L, monthly supplementation of 60,000 IU vitamin D_3_ for up to 5 years had little effect on reducing pain incidence and interference with daily activities (Rahman et al. 2023). Meanwhile, a 2024 Mendelian randomization study by Bassett E et al. refuted the universal benefits of VDS on pain from a genetic causal perspective (Bassett et al. 2024). In summary, existing evidence suggests that VDS holds certain potential as an adjunct therapy for NP, with relatively consistent short‐term benefits observed particularly in DPN and carpal tunnel syndrome. However, due to limitations such as small sample sizes, heterogeneous study designs, and insufficient follow‐up in current research, the evidence is still inadequate to incorporate VDS into routine clinical recommendations for NP.

### 6.2 A Review of Clinical Evidence for Vitamin D in CI

The role of vitamin D in CI has long been a research focus in the medical field. Currently, the clinical evidence shows a divergence between observational studies and RCTs, necessitating a systematic review and objective interpretation based on the hierarchy of evidence from different study designs. Evidence from observational studies is highly consistent: numerous cross‐sectional, case–control, and prospective cohort studies suggest that lower vitamin D levels are associated with poorer cognitive performance, CI, dementia, and an increased risk of AD. A dose–response meta‐analysis published by Huang et al. in 2025, which included 22 observational studies with a total of 53,122 participants, demonstrated that low vitamin D levels were associated with a 49% increased risk of dementia. The dose–response analysis indicated that for every 10 nmol/L increase in serum 25(OH)D, the risk of dementia decreased by 1.2% (Huang et al. 2025). A prospective cohort study conducted in 2024 based on the UK Biobank, including 269,229 participants aged 55–69 years with a 14‐year follow‐up, also found that compared to those with sufficient vitamin D levels, individuals with VDD had a 25%, 19%, and 24% increased risk of all‐cause dementia, AD, and vascular dementia, respectively. These findings further support that maintaining normal serum vitamin D levels or regularly taking supplements may have potential benefits in dementia prevention (Chen, Sha, et al. 2024). Fu et al.'s study further integrated 3935 brain MRI imaging phenotypes within the framework of Mendelian randomization (MR) to conduct mediation analysis, providing genetic evidence and imaging‐level insights into the neuroprotective mechanisms of Vitamin D. However, the mediation analysis indicated that white matter pathways mediated only a very small portion of the effect (7.08%), suggesting that Vitamin D's mechanisms may involve multiple pathways such as neuroinflammation regulation, calcium homeostasis maintenance, or vascular function protection (Fu et al. 2025). It is noteworthy that current large‐scale RCTs and meta‐analyzes consistently indicate that routine VDS does not provide significant benefits in improving cognition and mood among the general middle‐aged and elderly populations with sufficient vitamin D levels. The cognitive sub‐study of the VITAL trial showed that daily intervention with 2000 IU of vitamin D (median follow‐up of 2.8 years) did not slow overall cognitive decline in community‐dwelling older adults, and baseline vitamin D levels did not affect the outcomes. A slight cognitive maintenance benefit was observed only in the Black subgroup, but this finding requires further validation (Kang et al. 2021). Correspondingly, the VITAL‐DEP trial also confirmed that VDS failed to reduce the risk of depression or improve mood scores (Okereke et al. 2020). The same negative results were also replicated in the D‐Health study (Pham et al. 2023). Meanwhile, results from RCTs, which represent a higher level of evidence in the hierarchy of evidence‐based medicine, also show heterogeneity. The VitaMIND RCT published in 2025 enrolled 620 adults aged 50 and above with mild to moderate VDD and early CI, conducting a 24‐month supplementation intervention. The results demonstrated that VDS had no significant benefits on executive function, cognition, functionality, or well‐being at follow‐up. Secondary analyzes defining subgroups and per‐protocol analyzes likewise failed to show significant effects on any outcome measures (Corbett et al. 2025). Another systematic review and meta‐analysis encompassing 24 RCTs with a total of 7557 participants similarly demonstrated that VDS had a statistically significant but very small positive effect on overall cognitive function (Hedges' *g* = 0.128, *p* = 0.008), while no significant effects were observed in specific cognitive domains such as memory, executive function, attention, or information processing speed. Notably, further stratified analyzes suggested that the cognitive benefits of VDS were limited to certain populations, with relatively higher effect sizes observed in frail elderly individuals (*g* = 0.414) and those with baseline VDD (*g* = 0.480). Baseline nutritional status and population health characteristics appear to be key moderators influencing the intervention's effectiveness. Subgroup analyzes indicate that the benefits of supplementation are primarily confined to these specific subgroups (Chen, Cheng, et al. 2024).

In subjects with sufficient vitamin D levels, many supplementation trials yield negative results. This phenomenon mainly stems from multiple factors such as the body's homeostatic regulatory mechanisms and aspects of trial design. Once serum 25(OH)D reaches an adequate threshold (usually ≥ 50 nmol/L), additional supplementation is unlikely to further activate downstream biological effects or provide extra clinical benefits. Secondly, populations with adequate baseline nutrition inherently have a lower disease risk and a narrower window for observable effects, making it difficult for typical studies with limited sample sizes to detect statistically significant clinical outcomes. Moreover, most existing studies do not account for confounding factors like individual *VDR* gene polymorphisms, lifestyle habits, or inflammatory status, which ultimately hampers the ability of overall analyzes to identify potential benefits within subgroups. In summary, based on current clinical evidence, VDS is insufficient to be recommended as a cognitive enhancement therapy for the general population. Its clinical use should be strictly limited to specific groups with nutritional deficiencies. Furthermore, large‐scale, long‐term RCTs stratified by baseline vitamin D status are still needed to clarify the exact efficacy and optimal regimen of supplementation in deficient populations, as well as to verify the threshold hypothesis that supplementation is ineffective in individuals with sufficient levels.

### 6.3 Clinical Application Potential of Vitamin D in Comorbid NP and CI

Vitamin D exerts pleiotropic regulatory effects in both the central and PNS through genomic and non‐genomic pathways mediated by the VDR. VDR is widely expressed in the hippocampus, cortex, dorsal root ganglia, as well as various types of glial and immune cells. This distribution endows vitamin D with unique advantages in intervening in the comorbidity of NP and CI, specifically manifested in the following three dimensions. (1) Multi‐target Synergistic Regulation of Core Pathologies in Comorbidity. NP and CI share key pathological mechanisms such as neuroinflammation, neuroendocrine dysregulation, and brain network imbalance. Vitamin D, by binding to the widely distributed VDR on neurons, glial cells, and immune cells in both the central and PNS, participates in and modulates these multiple pathological processes, thereby achieving coordinated intervention of pain and cognitive function (Lupu et al. 2024; Carlberg and Velleuer 2024). (2) There is currently no consensus on Vitamin D screening, supplementation protocols, and long‐term monitoring for patients with NP and CI. Vitamin D supplementation strategies should be formulated comprehensively based on individual baseline Vitamin D status (such as baseline serum 25(OH)D levels), clinical characteristics (including age, comorbidities, renal function, and concomitant medications), and relevant clinical guidelines. A single dosage should not be regarded as universally applicable. For most general populations, a daily oral dose of 2000 IU is one of the commonly used doses to achieve and maintain adequate serum 25(OH)D levels, with overall good safety. However, considering individual differences and potential risks associated with long‐term use, dosage should be determined based on personalized assessments in clinical practice, with appropriate monitoring of serum levels and related indicators. Vitamin D–related hypercalcemia typically occurs only with prolonged intake of supraphysiological doses. It should be noted that 50,000 IU/day is a clinical therapeutic dose rather than a general safety threshold for the population; the tolerable upper intake level for adults is 4000 IU/day. Moreover, the risk of hypercalcemia is influenced by multiple factors such as duration of intake and underlying health conditions, and should not be generalized by a single dosage value (Efsa Panel On Nutrition, Novel Foods And Food Allergens (Nda) et al. 2023). Under standard supplementation regimens, the risk is negligible. In contrast, opioid analgesics carry risks of dependence and respiratory depression, while anticonvulsants and antidepressants often cause cognitive dulling, sedation, and other side effects. Some cognitive enhancers have limited efficacy and considerable individual variability. For elderly patients requiring long‐term management of comorbid conditions, vitamin D's safety advantage as a foundational adjunctive intervention is particularly prominent (Giustina et al. 2024; Bendotti et al. 2025). (3) Multiple Access Routes and High Cost‐effectiveness. Vitamin D is obtained from diverse sources, including endogenous synthesis through sunlight exposure, dietary intake, and oral supplements. Among these, oral formulations offer controllable dosing, are unaffected by seasonal or geographic variations, and have low production costs, effectively covering high‐risk groups such as those living in high‐latitude regions, individuals with darker skin tones, and obese populations. This high accessibility makes vitamin D a promising low‐cost and easily scalable public health intervention. In summary, vitamin D demonstrates a unique and pragmatic translational potential in the clinical adjunctive management of comorbid NP and CI, owing to its multi‐target pathological regulation, excellent long‐term safety, and high accessibility.

## 7 Conclusions and Future Outlooks

The comorbidity mechanisms of NP and CI are complex, and clinical intervention options are limited, posing a significant public health challenge in aging societies. Adequate levels of Vitamin D have been shown to be closely associated with favorable functional outcomes in the nervous, cardiovascular, and immune systems. Although the potential mechanisms of VDS in NP‐CI comorbidity have been preliminarily validated, several key controversies remain regarding its standardized clinical application as an adjunct therapy. Firstly, there is a lack of high‐quality evidence supporting the optimal dosage and administration regimen of Vitamin D in comorbid states. Existing studies are often limited to single diseases, whereas comorbidity frequently involves a more complex inflammatory microenvironment, metabolic disorders, and neural pathway damage, leading to significant differences in Vitamin D metabolism and requirements compared to single disease populations. Currently, there is a lack of large‐scale prospective clinical trials focusing on the synergistic improvement of clinical symptoms in comorbid patients, making it impossible to determine the specific dosage thresholds, administration routes, and ideal treatment duration for this population. Secondly, the mechanisms by which individual differences affect the efficacy of Vitamin D are not fully understood. It is known that polymorphisms in the VDR gene can influence the binding affinity of Vitamin D to its receptor and the efficiency of downstream signaling pathways, while hepatic and renal dysfunction can hinder the activation process of Vitamin D. Additionally, age‐related declines in metabolic capacity and the accumulation of Vitamin D in adipose tissue due to obesity can significantly alter its metabolic pathways and biological effects, resulting in substantial variability in patient responses to supplementation. Finally, research on Vitamin D combination therapy strategies is relatively weak, making it difficult to match the pathological characteristics of comorbidity with multiple mechanisms. The comorbidity of NP and CI involves multiple factors, such as neuroinflammation and neuroendocrine dysregulation, where single therapies often fail to achieve ideal outcomes. Theoretical applications of combining Vitamin D with first‐line analgesics, antidepressants, cognitive training, or other nutritional interventions could enhance treatment efficacy through multi‐target synergy. However, there are currently very few studies exploring the feasibility of such combination approaches. These controversies hinder the precise clinical translation and standardized application of Vitamin D.

Based on the potential roles of Vitamin D in NP and CI revealed by current research, as well as the associated controversies, future studies need to systematically explore its regulatory mechanisms in the context of comorbidity and promote standardized clinical applications from the following three perspectives: (1) Utilizing cutting‐edge technologies such as single‐cell sequencing, spatial transcriptomics, and multi‐omics integration, research should focus on elucidating the specific regulatory mechanisms of the Vitamin D/VDR signaling pathway in key brain regions such as the hippocampus and prefrontal cortex, as well as in the dorsal horn of the spinal cord. Additionally, using multimodal neuroimaging techniques, researchers should dynamically observe the effects of VDS on the functional connectivity and network properties of neural circuits related to NP and CI, thereby revealing the neurobiological basis for its simultaneous regulation of pain perception and cognition at the mechanistic level. (2) Conducting rigorous, large‐scale, long‐term RCTs targeting patients with NP‐CI comorbidity. The study design should emphasize the following aspects: stratified analysis based on polymorphisms in important metabolic genes such as VDR and CYP2R1 to identify potential responder populations; establishing cross‐population dose–response models to optimize personalized dosing regimens; and systematically evaluating the synergistic effects and safety of combining Vitamin D with medications (such as anti‐inflammatory analgesics and neuroprotective agents) and non‐pharmacological interventions (such as cognitive behavioral therapy and neuromodulation techniques). (3) Centered on precision medicine as the core framework, clinical evidence of Vitamin D in NP and CI should be explored through candidate biomarker screening and targeted subgroup enrichment. Firstly, it is essential to overcome the limitations of relying solely on serum 25(OH)D for nutritional assessment by incorporating factors such as VDR gene polymorphisms, cerebrospinal fluid Aβ/tau protein profiles, neuroinflammatory markers, and neural injury biomarkers. Secondly, high‐benefit target subgroups should be defined based on these biomarkers—for example, prioritizing individuals with baseline VDD or insufficiency, carriers of specific susceptible VDR genotypes, and patients with NP and early CI accompanied by low‐grade neuroinflammation. Subsequent study designs should adopt biomarker‐driven enrichment strategies, using standardized rating scales and dynamic changes in biomarkers as endpoints to systematically validate the targeted effects of Vitamin D in pain relief and neuroprotection, thereby promoting its transition from broad supplementation to subgroup‐specific precision intervention. (4) Based on high‐quality clinical evidence, developing expert consensus or clinical guidelines for VDS applicable to NP‐CI comorbidity, clarifying the target populations, recommended dosages, monitoring indicators, and safety warnings. Strengthening health education aimed at the public and healthcare professionals to enhance awareness of the relationship between Vitamin D nutritional status and neuropsychiatric comorbidities. Furthermore, leveraging wearable devices and AI‐assisted digital health platforms to achieve dynamic monitoring and personalized management of Vitamin D levels in high‐risk populations and patients, facilitating early screening and precise interventions to prevent complications resulting from severe deficiency.

In summary, Vitamin D supplementation may serve as a potential adjunctive strategy for patients with comorbid NP and CI; however, given the limited current evidence and the lack of high‐quality randomized controlled trials, further well‐designed clinical studies are needed before its clinical application can be recommended. It is hoped that through the deep integration of mechanistic basic research and clinical practice, the regulatory networks of Vitamin D in NP‐CI comorbidity can be further elucidated, and biomarker‐based personalized supplementation strategies established. This would position Vitamin D as an effective nutritional adjunct in the comprehensive management of patients with NP‐CI comorbidity, contributing to improved overall prognosis and quality of life.

## Author Contributions

**Nan Zhao:** conceptualization, writing – original draft, writing – review and editing. **Yang Yang:** visualization, writing – review and editing. **Wanqiu Yu:** writing – review and editing, visualization. **Yuhang Zhu:** conceptualization, writing – review and editing. **Zhaoqiong Zhu:** writing – review and editing, funding acquisition.

## Funding

This work was supported by the Guizhou Provincial Basic Research Program (Natural Science) [QKHJC(2026)002‐90], Zunyi Science and Technology Cooperation Fund (HZ2026‐367), Guizhou Provincial Science and Technology support plan project, qiankehe support [2025] general 334, 2025 Science and Technology Fund Project of Guizhou Provincial Health and Welfare Commission (gzwkj2025‐381).

## Conflicts of Interest

The authors declare no conflicts of interest.

## Data Availability

Data sharing is not applicable to this article as no new datasets were generated or analyzed during the current study.
